# Modulation of Standing Spin Waves in Confined Rectangular Elements

**DOI:** 10.3390/ma17102404

**Published:** 2024-05-16

**Authors:** Milad Jalali, Qian Chen, Xuejian Tang, Qingjie Guo, Jian Liang, Xiaochao Zhou, Dong Zhang, Zhaocong Huang, Ya Zhai

**Affiliations:** 1 Key Laboratory of Quantum Materials and Devices of Ministry of Education, School of Physics, Southeast University, Nanjing 211189, China; 233189936@seu.edu.cn (M.J.); qchen2022@seu.edu.cn (Q.C.); 220212158@seu.edu.cn (X.T.); 230208202@seu.edu.cn (Q.G.); 230189565@seu.edu.cn (J.L.); 230159126@seu.edu.cn (X.Z.); hzc28@seu.edu.cn (Z.H.); 2 School of Physical Science and Information Technology, Liaocheng University, Liaocheng 252059, China

**Keywords:** spin waves, magnetisation dynamics, magnetic thin films, micromagnetic simulations

## Abstract

Magnonics is an emerging field within spintronics that focuses on developing novel magnetic devices capable of manipulating information through the modification of spin waves in nanostructures with submicron size. Here, we provide a confined magnetic rectangular element to modulate the standing spin waves, by changing the saturation magnetisation (*M*_S_), exchange constant (*A*), and the aspect ratio of rectangular magnetic elements via micromagnetic simulation. It is found that the bulk mode and the edge mode of the magnetic element form a hybrid with each other. With the decrease in *M*_S_, both the Kittel mode and the standing spin waves undergo a shift towards higher frequencies. On the contrary, as *A* decreases, the frequencies of standing spin waves become smaller, while the Kittel mode is almost unaffected. Moreover, when the length-to-width aspect ratio of the element is increased, standing spin waves along the width and length become split, leading to the observation of additional modes in the magnetic spectra. For each mode, the vibration style is discussed. These spin dynamic modes were further confirmed via FMR experiments, which agree well with the simulation results.

## 1. Introduction

The study of spin waves has garnered considerable interest in recent years due to its wide-ranging implications in industries such as information processing and emerging areas like quantum computing [1,2,3]. The emerging field for applied spin waves is known as magnonics [4,5]. Utilising spin waves as carriers of information can avoid the inherent constraints of modern electronics, such as energy waste caused by Joule heating due to Ohmic losses [6]. It has been shown that spin wave excitation in nano-systems has potential applications in multiplexers, logic gates, transistors, and magnonic crystals, among other technologies [7,8,9,10,11].

The fundamental components of magnonic devices consist of patterned, magnetic thin films [12]. The magnetization dynamics of a magnetic material undergoes significant changes as its dimension is reduced, leading to the excitation of quantized spin wave (SW) modes due to the non-uniform internal field [13].

Ni_80_Fe_20_, commonly known as Permalloy or Py, is a magnetic material highly regarded for its exceptional properties. It features a high Curie temperature, low coercivity, and minimal anisotropies, making it a premier selection due to its significant saturation magnetization (*M*_S_) and low damping factor (*α*). These attributes can be tailored through the introduction of heavy metal [14] and rare-earth elements [15], which we have successfully demonstrated in our previous experimental investigations. These properties are advantageous as they ensure long spin wave lifetimes, increase spin wave frequencies and velocities, and provide additional thermostability [16]. It can also be easily deposited and nanostructured. Thus, patterning and structural design on Py films has become one of the research hotspots in magnonics. The lateral spin wave excitation in patterned elements has been extensively investigated since 1998 using the BLS approach [17,18], ultrafast optical method [19], FMR [20], and so on.

However, in such investigations, the effects of structural parameters on the excited spin waves in magnetic elements is limited. It is known that the interactions between elements could be ignored when the element’s distance is larger than 1 micron, while earlier works focused on patterns with small element separation and mostly focused on the geometries of strips, circles, and circular rings [21,22]; to the best of our knowledge, reports on systematic studies of size and shape have rarely been seen. More importantly, with the development of simulation software like Oriented Micromagnetic Framework (OOMMF, version 1.2) and Comsol Multiphysics (version 6), calculations are more accurate than earlier studies that used approximation formula derivation. Consequently, it is crucial to achieve a comprehensive understanding and precise control of spin dynamics within magnetic elements [23,24,25].

Micromagnetic simulation act as a bridge between theoretical spintronics and experiments. It can investigate related phenomena like exchange interaction in specific structures [26] and spin dynamic modes resulting from the magnetisation precession. In this work, we systematically study the spin dynamics in rectangular magnetic elements using micromagnetic simulation software COMSOL (version 6) and OOMMF (version 1.2). We selected rectangular elements for our study based on their non-uniform demagnetization fields, which facilitate the excitation of various spin wave modes, as well as the structural simplicity that enables systematic investigation and control over spin waves through the adjustment of key parameters. As a result, different kinds of spin dynamic modes, including the Kittel mode and standing spin waves, were observed. Furthermore, effective manipulation of spin waves was achieved by deliberately adjusting key parameters such as saturation magnetisation (*M*_S_), the exchange constant (*A*), and the aspect ratio of rectangular magnetic elements. The substantiation of these spin dynamic modes, as well as their response to changes in the aspect ratio, is reinforced by experimental results obtained through FMR measurements, which exhibit a close agreement with the simulation results. Our study provides a solid foundation for the modulation and harnessing of spin waves, serving as a key reference for their application in magnonic devices.

## 2. Materials and Methods

Micromagnetic simulations of spin dynamics in rectangular magnetic elements were studied in the frequency domain using the COMSOL Multiphysics Micromagnetic Module [26]. In addition, OOMMF-based Python programming is also used to study the dispersion of these spin dynamics in the time domain, which is further converted into the frequency domain via Fast Fourier Transform (FFT) [27]. The spin dynamics are defined using the LLG equation [28,29]:

(1) $$\dot{\vec{m}}=-\gamma \vec{m}\times {\vec{H}}_{eff}+\alpha \vec{m}\times \dot{\vec{m}}$$

where $\vec{m}$ is the unit vector of magnetization, γ denotes the gyromagnetic ratio, and α is the Gilbert damping constant. ${\vec{H}}_{eff}=H_{ex}+H_{d}+H_{ext}+h$ is the effective field, including the exchange field $H_{ex}=A\nabla^{2}\vec{m}$, demagnetizing field $H_{d}$, in-plane alternating excitation magnetic field ***h***, and the external field $H_{ext}$, which is applied perpendicular to the film surface. *A* represents the exchange constant for the ferromagnetic material. The parameters for micromagnetic simulations are listed in Table 1. The values of *M*_S_ and *α* were determined based on experimental data reported in previous studies on Py-based films [14,15]. We focus on the dynamic behaviour of magnetization, which can be separated into static and dynamical parts, namely $\vec{m}=m_{0}+\delta m$***,*** where $m_{0}$ is the spatial profile of the static magnetization background at equilibrium and δm is the dynamical excitation upon the static background which is perpendicular to $m_{0}$. The LLG equation can be described in the context of the frequency domain as follows:

(2) $$i\omega \delta m=-\gamma m_{0}\times \delta {\vec{H}}_{eff}-\gamma \delta m\times {\vec{H}}_{0}^{eff}+i\omega \alpha m_{0}\times \delta m.$$

We employed the COMSOL Frequency Domain Micromagnetics module to compute the eigenvalues of the magnetization precession process, leading to the derivation of a frequency spectrum and the spatial profile of the magnetization precession. The dipole field distribution is established by concurrently utilizing the micromagnetics module and the AC/DC module within COMSOL Multiphysics. This is achieved by solving Maxwell’s equations and the LLG equation in a coupled manner. Furthermore, in our simulations using OOMMF, we initiated the relaxation of magnetic moments to their equilibrium positions before inducing magnetization precession.

The main sample structure for the experimental study was Ta (5 nm)/Py (10 nm)/Ta (5 nm), where the upper and lower layers of Ta served as a buffer and protection layer. Films were deposited on Si/SiO_2_ substrates at room temperature via magnetron sputtering under a background chamber pressure of about 1.5 × 10^−5^ pa to guarantee high-quality samples. The argon pressure was kept at 0.3 Pa to start magnetron sputtering. Then, rectangular arrays on the submicron scale were prepared using electron beam lithography. The width *w* of the element in the array was 300 nm, and the rectangular ratios were 1, 2, and 4. The structure properties were characterised using a scanning electron microscope (SEM), while the dynamic magnetism properties of the samples were studied using cavity FMR measurements.

## 3. Results and Discussion

**Figure 1:**
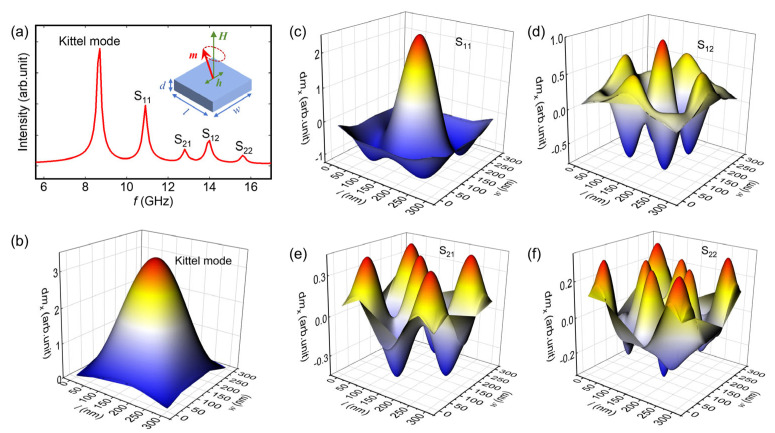
(**a**) Frequency domain magnetic spectra of a square element with a geometrical size of 300 nm × 300 nm × 10 nm. Inset shows the model used in simulation. The red arrow signifies the magnetization, while the single-direction green arrow denotes the magnetic field, and the double-headed green arrow represents the microwave magnetic field. The spatial distribution of the amplitude of magnetisation precession of (**b**) the Kittel mode, (**c**) S_11_ (**d**) S_12_ (**e**) S_21_, and (**f**) S_22_. The color indicates the component of magnetic moment precession in the *x*-direction from minimum value (blue) to maximum value (red).

Figure 1a illustrates the magnetic spectrum with the frequency domain of a sample with a geometrical size of 300 nm × 300 nm × 10 nm obtained via COMSOL simulation. The intensity of the spectra is directly proportional to the magnitude of the magnetic moment precession. The simulation includes an applied magnetic field that is perpendicular to the surface of the film, with an intensity of 12 k Oe. The sample is enclosed within a vacuum sphere with a radius of 2000 nm to provide a demagnetising field. A series of multi-peaks are observed from the magnetic spectrum, as shown in Figure 1a. Based on the peak intensity, we have initially identified the presence of at least three separate sets of modes. In order to gain a better understanding of the underlying mode structure, we have focused our attention on the spatial distribution of the amplitude of magnetization precession at various frequencies corresponding to Figure 1a. It indicates that the peak with the highest intensity represents the mode in which the majority of magnetisations within the element are undergoing uniform precession, commonly referred to as the Kittel mode [30], as shown in Figure 1b. The spatial profiles of modes S_11_ and S_12_ are presented in Figure 1c,d, respectively. It is evident that under these excitation modes, there exists a discernible contrast in the magnitudes of magnetization precession between the boundary and the interior regions of the element. The higher demagnetising field at the boundary compared to the interior region leads to an inhomogeneous distribution of the effective field within the element, causing this discrepancy. The hybridised spin precession modes S_11_ and S_12_, arising from the coupling between the boundary magnetization and the bulk magnetisation, highlights the complex interplay between the boundary and the bulk in this system. Additionally, another set of spin precession modes, denoted as S_21_ and S_22_, have been observed. Their spatial profiles are illustrated in Figure 1e,f. We have identified that these precession modes arise from the vector sum of standing spin waves along the two sides of the square. Notably, the standing spin waves on both sides exhibit identical mode numbers owing to the equivalent dimensions of the element in terms of width and length, such as (3,3) for S_21_ and (5,5) for S_22_. Since the magnetic field is perpendicular to the film plane, the standing spin wave with a different mode propagates along the film [31,32].

**Figure 2:**
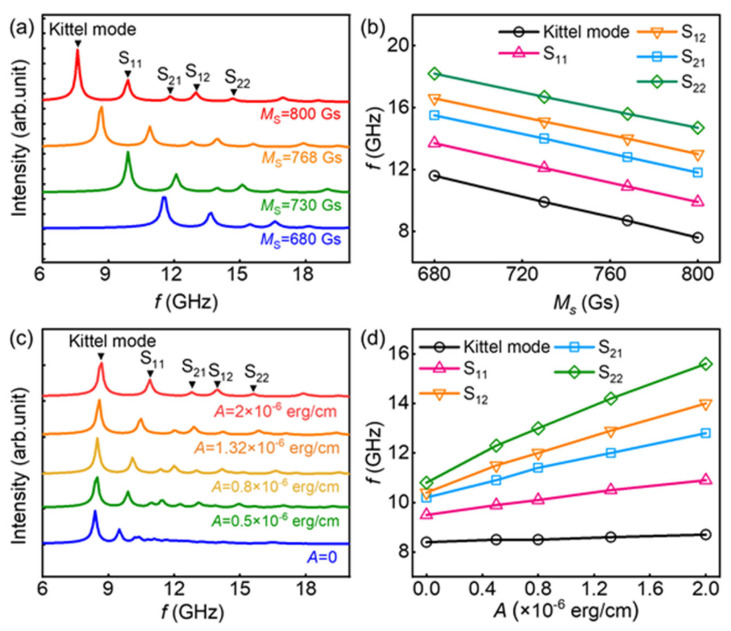
(**a**) Effect of changing *M*_S_ on the frequency domain magnetic spectrum of the ferromagnetic element with a geometrical size of 300 nm × 300 nm × 10 nm. The external magnetic field is 12 k Oe. (**b**) *M*_S_ dependence of the intrinsic frequency of different modes. (**c**) Effect of changing *A* on the frequency domain magnetic spectrum. (**d**) *A* dependence of the intrinsic frequency of different modes.

After establishing the mode structures, we investigate the influence of material and structural parameters on their modulations. Figure 2a displays the frequency domain magnetic spectrum of a square element with dimensions of 300 nm × 300 nm × 10 nm, obtained via simulation at various values of saturation magnetisation *M*s. The exchange constant is kept as *A* = 2 × 10^−6^ erg/cm. At various values of *M*s (680, 730, 768, and 800 Gs), the mode structures observed from the spectrum remains largely unchanged. However, as the *M*s increases, the entire frequency spectrum shifts towards lower frequencies.

The reason for this is that a higher saturation magnetisation necessitates a stronger magnetic field to achieve saturation. Since the external magnetic field in our simulation is fixed, the corresponding intrinsic frequency for each precession mode becomes smaller according to the Kittel equation [33,34,35], which establishes an inverse relationship between magnetic field and frequency. The variations in intrinsic frequencies (peak positions) for different modes along with *M*_S_ are depicted in Figure 2b, which evidently shows a similar nearly linear decreasing tendency with increasing *M*_S_.

Subsequently, we direct our attention to the influence of the exchange constant *A* on spin dynamic modes. In Figure 2c, the frequency domain magnetic spectra of a square element (300 nm × 300 nm × 10 nm) with an *M*_S_ of 768 Gs are presented. As we can see in the figure, the main resonance peak does not change much. With the increase in the exchange constant, the higher-order modes quickly move towards the higher frequencies, and the resonance peak separation becomes larger. When *A* = 0, the spectrum exhibits only the peaks associated with the boundary modes, while the previously occurring region of bulk spin standing waves becomes disordered. This can be attributed to the prevailing long-range magnetic dipole interactions among the magnetic moments, making it challenging to excite spin standing waves of shorter wavelengths. Since the demagnetising field is concentrated at the boundaries, inhomogeneous precession at those locations can be excited. The extracted intrinsic frequencies for different modes as a function of *A* are depicted in Figure 2d. It is observed that the various types of precession modes exhibit distinct trends with varying *A*. We note that the intrinsic frequency of the Kittel mode remains relatively unaffected by changes in *A*. Conversely, the intrinsic frequencies of S_11_, S_12_, S_21_, and S_22_ exhibit nearly linear increases with rising *A*; the distinct responses of these various modes to *A* are determined by the mode types. The Kittel mode displays negligible phase difference between the precessing moments as a result of the quasi-uniform precession of magnetic moments. As a consequence, the exchange interaction has little influence on the Kittel mode’s intrinsic frequency. On the other hand, S_21_ and S_22_ arise from spin waves dominated by the exchange interaction, with their precession frequencies directly proportional to the exchange coefficient and determined by the square of the wave vector. Consequently, as the mode number increases, so does the wave vector, leading to a larger response to *A*. S_11_ and S_12_, on the other hand, are hybrid modes formed through the coupling of bulk spin waves and boundary spin waves [23,36,37]. Their precession frequencies are jointly determined by the exchange interaction and magnetic dipole interaction [23,38]. Due to the confinement of the element’s dimensions to 300 nm, these non-uniform precessions are predominantly governed by the exchange interaction, with a relatively weaker contribution from the magnetic dipole interaction. As the mode number increases, the phase difference between adjacent magnetic moments also increases, leading to a greater response to the exchange interaction.

**Figure 3:**
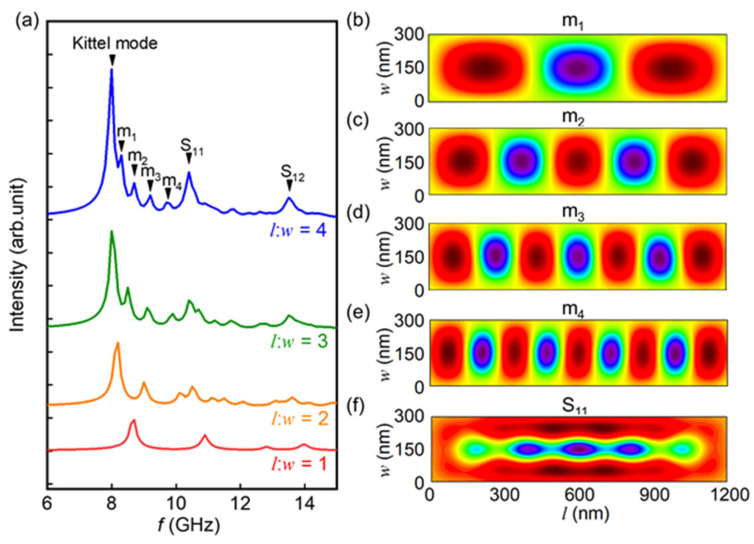
(**a**) Simulated frequency spectrum of the rectangular unit with aspect ratios of 1, 2, 3, and 4. The width is fixed to 300 nm and the length varied as 300, 600, 900, and 1200 nm. Spatial distribution of the magnetization precession intensity of (**b**) m_1_, (**c**) m_2_, (**d**) m_3_, (**e**) m_4_, and (**f**) S_11_ for the sample with an aspect ratio of 4 labelled in (**a**). The color indicates the component of magnetic moment precession in the *x*-direction from minimum value (blue) to maximum value (red).

Next, we proceed to explore the impact of the shape of the magnetic element on the excitation of spin dynamic modes. Figure 3a depicts the simulated spectrum of a rectangular element with a width of 300 nm, a thickness of 10 nm, and aspect ratios of 1, 2, 3, and 4 (corresponding to lengths of 300, 600, 900, and 1200 nm, respectively). The *M*_S_ and *A* of the material are set as 2 × 10^−6^ erg/cm and 768 Gs, respectively. In contrast to the scenario with an aspect ratio of 1, it has been observed that, when the length and width of the magnetic element are unequal, numerous additional peaks appear between the Kittel mode and the original S_11_ mode. The number of these additional peaks increases with the increment of the aspect ratio. This indicates a modulation in the mode structure of spin precession with varying aspect ratios. In order to elucidate the changes in the mode structure, we examined the spatial distribution of the magnetization precession intensity. Figure 3b–f presents the spatial distributions of several modes (m_1_, m_2_, m_3_, m_4_, and S_11_, as labelled in Figure 3a) within the element with an aspect ratio of 4. We find that, when the length and width of the unit cell are unequal, the mode numbers of the standing spin waves excited along the boundaries are different, leading to the formation of distinct hybrid states. For instance, the satellite peak m_1_ arises from the superposition of the standing spin wave excited along the shorter dimension with mode number 1 and those along the longer dimension with mode number 3, denoted as m(1,3). The satellite peak m_2_ results from the superposition of the spin waves excited along the shorter dimension with mode number 1 and those along the longer dimension with mode number 5, denoted as m(1,5). Similarly, m_3_ and m_4_ represent m(1,7) and m(1,9), respectively. Meanwhile, the peak corresponding to S_11_ aligns with the bulk-edge hybridised mode observed previously in the element with an aspect ratio of 1.

**Figure 4:**
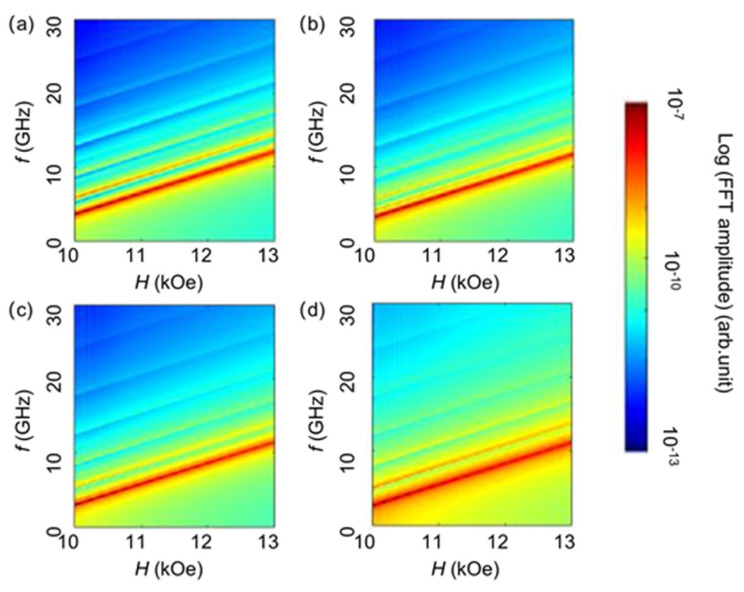
The dispersion relations of spin precession modes in elements with aspect ratios of (**a**) 1, (**b**) 2, (**c**) 3, and (**d**) 4, respectively.

In order to facilitate further investigations into ferromagnetic resonance, we conducted simulations to study the dispersion relations of spin precession modes in elements with different aspect ratios, and the results are presented in Figure 4 [39]. As the external magnetic field increases, the resonant frequencies of different modes become larger. At a fixed frequency, the Kittel mode requires the highest magnetic field for precession, while the external magnetic field required for standing spin wave precession is smaller and is dependent on the mode number. The higher the mode number, the smaller the corresponding magnetic field required.

**Figure 5:**
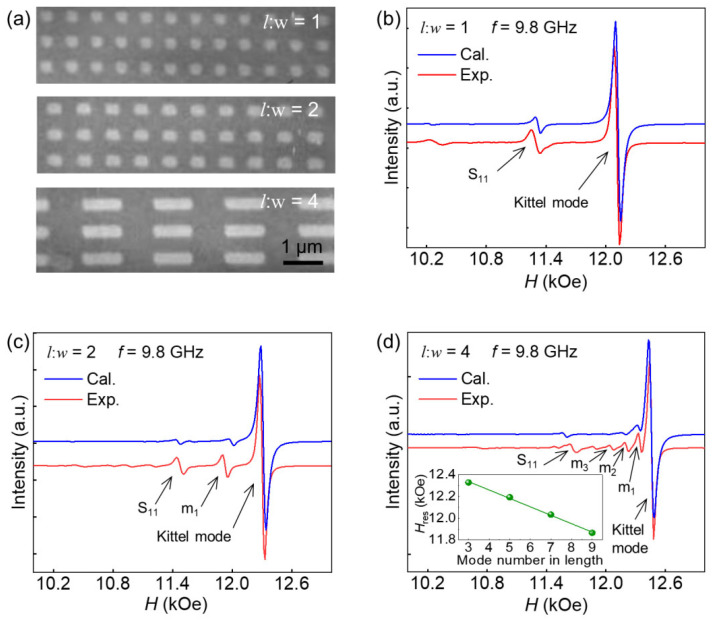
(**a**) Scanning electron microscopy images of three different ratios (1, 2, and 4) of a patterned thin film. The ferromagnetic resonance spectra of the elements with aspect ratios (**b**) 1, (**c**) 2, and (**d**) 4. The blue lines are the simulation curves, and the red lines are the experimental curves in the field range from 10 to 13 k Oe and the fix frequency of 9.8 GHz. The insert shows the relationship between the resonance field and the mode number in length obtained from experimental results.

We next performed the experimental investigations on an array of magnetic elements with different aspect ratios; Figure 5a presents the scanning electron microscope (SEM) images of the samples, showcasing their excellent structural uniformity. Through cavity ferromagnetic resonance measurements at a fixed frequency (9.8 GHz), the precession intensity of magnetic moments as a function of magnetic field is achieved, as illustrated by red lines in Figure 5b–d. In order to better compare the experimental results with the micromagnetic simulation results, we first extracted the precession spectra of magnetic moments at 9.8 GHz and transferred the frequency domain to magnetic field domain from Figure 4, as represented by the blue curves in Figure 5b–d. By modulating the simulation parameters *M*_S_ and *A*, the observed agreement between the theoretical simulation curves and the experimental curves indicates the reliability of our simulation results and their potential for guiding experimental investigations. By conducting a comparative analysis of the spectra and integrating them with the dispersion relationships depicted in Figure 4, we are able to illustrate the various precession modes that manifested in the experimental data. We observed that, apart from the Kittel mode, samples with an aspect ratio of 1 exhibited a discernible resonance peak corresponding to the bulk-edge hybridisation mode S_11_. Furthermore, in samples with aspect ratios of 2 and 4, in addition to the observed bulk-edge hybridised modes, we also discerned the presence of spin standing wave modes such as m_1_, m_2_, m_3_, and m_4_. These modes and their respective resonance field positions have been denoted in Figure 5b–d for reference. According to the experimental results, we have extracted the relationship between the mode number of the spin wave in the long-edge direction and the corresponding resonance field, as shown in Figure 5d. The resonance field decreases linearly with the increase in mode number, which is different from the square relationship between the mode number of the spin wave propagating in the thickness direction and the resonance field.

## 4. Conclusions

In conclusion, we have conducted a comprehensive investigation into the spin dynamic modes in magnetic rectangular elements and proposed practical strategies for their effective modulation. The micromagnetic simulations confirmed that the mode structure predominantly relies on the structural parameters of the rectangular element, and the intrinsic frequencies of different modes can be manipulated by adjusting material properties such as *M*_S_ and *A*. When the length and width of the rectangular element are equal, three distinct spin precession modes are observed: the Kittel mode, a hybrid mode combining edge and bulk spin wave modes, and the superimposed bulk standing spin wave modes. The Kittel mode displays a decrease in intrinsic frequency with increasing *M*_S_ under a fixed magnetic field, while it remains almost unaffected by changes in *A*. Conversely, the intrinsic frequencies of spin-wave-related precession modes decrease with increasing *M*_S_ but increase with *A*. The behaviour of various modes in response to *M_S_* displays a similar trend, while spin wave modes with higher mode numbers demonstrate a more significant reliance on *A*. When the length and width of the rectangular element are not equal, standing spin waves with different mode numbers emerge in both the length and width directions, leading to the presence of several extra spin wave modes. The number of these modes grows as the aspect ratio of the rectangle rises. Through ferromagnetic resonance experimental studies, we have successfully verified the existence of these distinct precession modes. The experimentally determined modes are consistent with the micromagnetic simulations’ predictions, demonstrating a correlation between mode variations and aspect ratio changes. Our research establishes a robust basis for controlling and utilising spin waves, offering valuable insights for their implementation in magnonic devices.

## Figures and Tables

**Table 1:** Parameters for micromagnetic simulations (*d*: thickness, *w*: width, *l*: length, *γ*: gyromagnetic ratio, and *α*: Gilbert damping).

Fixed Parameters				Variable Parameters		
*d* (nm)	*w* (nm)	*γ* (*GHz*/*kOe*)	*α*	*l* (nm)	*M*_S_ (Gs)	*A* (×10^−6^ erg/cm)
10	300	17.59	0.012	300, 600, 900, 1200	680, 730, 768, 800	0, 0.5, 0.8, 1.32, 2

## Data Availability

Data are contained within the article.
